# Do malaria ookinete surface proteins P25 and P28 mediate parasite entry into mosquito midgut epithelial cells?

**DOI:** 10.1186/1475-2875-4-15

**Published:** 2005-02-25

**Authors:** Luke A Baton, Lisa C Ranford-Cartwright

**Affiliations:** 1 Division of Infection and Immunity, Institute of Biomedical and Life Sciences, Joseph Black Building, University of Glasgow, Glasgow, G12 8QQ, UK

## Abstract

**Background:**

P25 and P28 are related ookinete surface proteins highly conserved throughout the *Plasmodium *genus that are under consideration as candidates for inclusion in transmission-blocking vaccines. Previous research using transgenic rodent malaria parasites lacking P25 and P28 has demonstrated that these proteins have multiple partially redundant functions during parasite infection of the mosquito vector, including an undefined role in ookinete traversal of the mosquito midgut epithelium, and it has been suggested that, unlike wild-type parasites, Dko P25/P28 parasites migrate across the midgut epithelium *via *an intercellular, rather than intracellular, route.

**Presentation of the hypothesis:**

This paper presents an alternative interpretation for the previous observations of Dko P25/P28 parasites, based upon a recently published model of the route of ookinete invasion across the midgut epithelium. This model claims ookinete invasion is intracellular, with entry occurring through the lateral apical plasma membrane of midgut epithelial cells, and is associated with significant invagination of the midgut epithelium localised at the site of parasite penetration. Following this model, it is hypothesized that: (1) a sub-population of Dko P25/P28 ookinetes invaginate, but do not penetrate, the apical surface of the midgut epithelium and thus remain within the midgut lumen; and (2) another sub-population of Dko P25/P28 parasites successfully enters and migrates across the midgut epithelium *via *an intracellular route similar to wild-type parasites and subsequently develops into oocysts.

**Testing the hypothesis:**

These hypotheses are tested by showing how they can account for previously published observations and incorporate them into a coherent and consistent explanatory framework. Based upon these hypotheses, several quantitative predictions are made, which can be experimentally tested, about the relationship between the densities of invading Dko P25/P28 ookinetes in different regions of the midgut epithelium and the number of oocyst stage parasites to which these mutant ookinetes give rise.

**Implications of the hypothesis:**

The recently published model of ookinete invasion implies that Dko P25/P28 parasites are greatly, although not completely, impaired in their ability to enter the midgut epithelium. Therefore, P25 and/or P28 have a novel, previously unrecognized, function in mediating ookinete entry into midgut epithelial cells, suggesting that one mode of action of transmission-blocking antibodies to these ookinete surface proteins is to inhibit this function.

## Background

P25 and P28 are related major ookinete surface proteins under consideration as candidates for inclusion in transmission-blocking vaccines [1-4]. Consequently, the expression [5-18], localisation [8,12,17-24] and function [21,25-29] of these molecules, together with the effect on parasite development of specific antibodies against them [6,8,21,22,24,30-35], have been extensively studied in a range of malaria parasite species.

P25 and P28 are structurally similar proteins, highly conserved throughout the *Plasmodium *genus [11,12,31,35-43], which contain four epidermal growth factor-like domains [36], putatively involved in cell-cell and/or cell-matrix interactions [21,25,26,28,29], that are expressed throughout the early life-cycle stages of the malaria parasite within the mosquito vector – from the macrogamete through to the oocyst stage [8,12,17-24]. P25 and P28 are located on the parasite surface, from which they are shed during ookinete gliding motility and traversal of the mosquito midgut epithelium [19-21,44,45]. The conservation of sequence, expression and location suggests that P25 and P28 have functionally equivalent roles in diverse malaria parasite species.

Previous research using transgenic *Plasmodium berghei *rodent malaria parasites lacking P25 and P28 demonstrated that these proteins have multiple and partially redundant functions during parasite infection of the mosquito vector [26,27]. Although Dko P25/P28 *P. berghei *parasites exhibit greatly reduced levels of oocyst infection compared to wild-type or Sko P25/P28 parasites, ookinetes lacking both P25 and P28 are still able to cross the midgut epithelium and establish oocyst infections [27]. Wild-type *P. berghei *ookinetes migrate intracellularly through the midgut epithelium causing significant damage to invaded midgut epithelial cells [44-48], which subsequently exhibit distinct morphological abnormalities [44-48], including loss of microvilli [44,45], protrusion into the midgut lumen [44,45,48] and up-regulation of molecules implicated in mosquito immune responses such as NOS [44,49] and SRPN10 [45,50]. Furthermore, P28 is found on the apical surface, and within the cytoplasm, of these abnormal midgut epithelial cells suggesting release/secretion from penetrating parasites during their intracellular migration [44,45]. Dko P25/P28 ookinetes have also been found deep within the midgut epithelium [27,45]. Initially, these parasites were suggested to be retarded in their transit through the midgut epithelium and killed by the epithelial cell defence reactions triggered by wild-type parasites [27]. Recently, however, Dko P25/P28 parasites were observed apparently deep within the midgut epithelium between morphologically normal midgut epithelial cells [45]. These midgut epithelial cells did not exhibit the abnormal characteristics typically associated with invasion by wild-type ookinetes, such as protrusion into the midgut lumen and up-regulation of SRPN10 [44,45,48]. Consequently, these Dko P25/P28 parasites were proposed to be migrating through the midgut epithelium *via *a solely intercellular route [45]. However, a recently published model of ookinete invasion across the mosquito midgut epithelium [51] suggests an alternative interpretation for the previously published observations of Dko P25/P28 parasites.

## Presentation of the hypothesis

### A unified model of the route of ookinete invasion across the mosquito midgut epithelium

**Figure 1:**
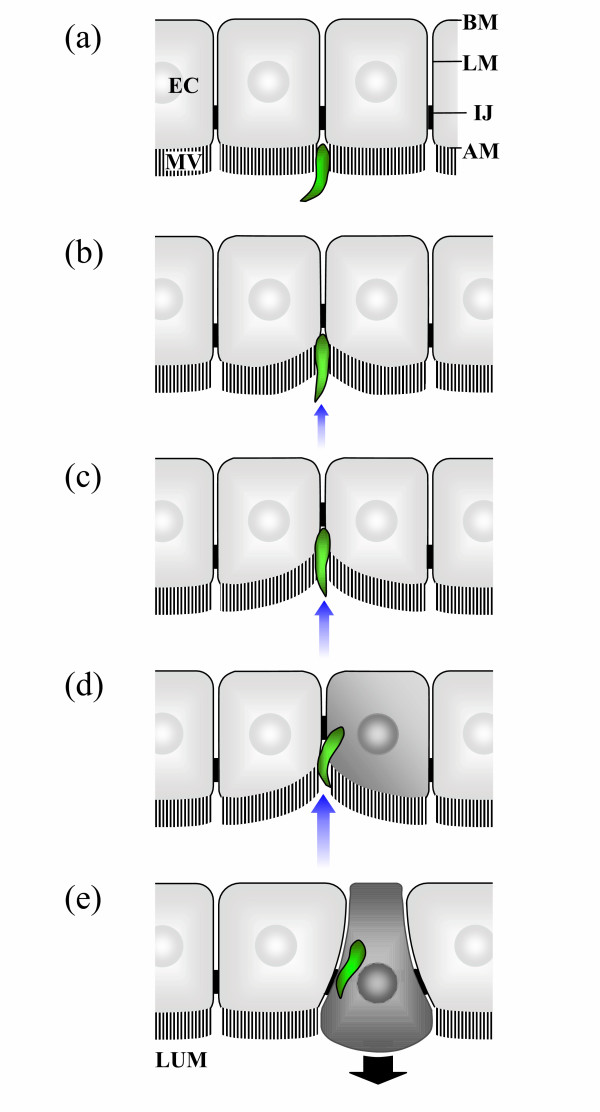
**A general model of ookinete entry into the mosquito midgut epithelium. **(a) Ookinetes (shown in green) enter the apical surface of the midgut epithelium, through the microvillar brush border (MV), where the lateral membranes (LM) of adjacent epithelial cells (EC) converge [47,51,52]. (b-c) Ookinete movement into the midgut epithelium causes substantial localized invagination of the latter (indicated by small blue arrows) [52,57]. (d) Ookinetes subsequently enter midgut epithelial cells through the lateral apical membrane immediately adjacent to the site where the intercellular junctions (IJ) begin [47,51,52]. (e) The ookinete proceeds intracellularly towards the basal membrane (BM) of the invaded midgut epithelial cell which exhibits morphological abnormalities including protrusion (indicated by large black arrow) into the midgut lumen (LUM) [44–48,52,54,55].

The route of ookinete migration across the midgut epithelium of the mosquito vector has long been controversial [51]. The major argument in the literature has been whether ookinete invasion is either solely intercellular between, or intracellular through, midgut epithelial cells [51]. Recently, an attempt has been made to unify the apparently conflicting literature and integrate it with other recent observations [44,47,52] in order to provide a single general model of the route of ookinete invasion across the midgut epithelium applicable to diverse malaria parasite and mosquito vector species [51]. Subsequent observations of ookinete invasion of the midgut epithelium *in vivo *support this model [48]. According to the model, ookinete entry into the midgut epithelium is initially intracellular, occurring through the lateral apical plasma membrane of midgut epithelial cells (Figure 1) [47,51,52]. Significantly, ookinete entry into midgut epithelial cells is often associated with substantial local invagination of the midgut epithelium [52], an observation supported by re-interpretation of previously published images (Figure 2 in Ref. [19] and Figure 5 in Ref. [53]). Ookinetes pass intracellularly through one or more midgut epithelial cells, causing significant damage similar to that described for wild-type *P. berghei *ookinetes [44-48,51,52,54,55]. Subsequently, ookinetes exit invaded epithelial cells into the basolateral extracellular space between adjacent midgut epithelial cells [48,52,56], migrate intercellularly to the basal surface of the midgut epithelium and transform into oocyst stage parasites [51].

**Figure 2:**
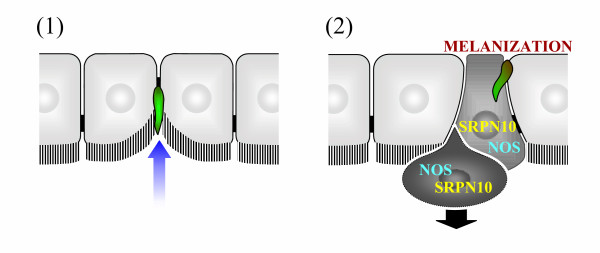
**Dko P25/P28 *P. berghei *ookinete invasion of the midgut epithelium. **The unified model of the route of ookinete invasion across the mosquito midgut epithelium (Figure 1) [51] implies that there are two sub-populations of Dko P25/28 parasites: (1) a major sub-population of Dko P25/28 ookinetes (shown in green) unable to penetrate midgut epithelial cells, which remain extracellular within the midgut lumen, embedded against the invaginated apical surface of the midgut epithelium (indicated by small blue arrow); and (2) a minor sub-population of Dko P25/28 ookinetes able to penetrate midgut epithelial cells, causing activation of mosquito immune responses and protrusion of invaded midgut cells, in a manner similar to wild-type parasites. Whether the latter parasites migrate through multiple adjacent midgut epithelial cells (as shown) is uncertain.

### Significance of the unified model for understanding Dko P25/P28 *P. berghei* ookinete invasion

Following the model of ookinete invasion of the midgut epithelium outlined above (and Figure 1), two hypotheses about Dko P25/P28 *P. berghei *parasite infection of the mosquito vector are proposed. First, some Dko P25/P28 ookinetes invaginate, but are unable to penetrate, the apical surface of the midgut epithelium. Second, other Dko P25/P28 parasites are able to successfully enter and migrate across the midgut epithelium *via *an intracellular route, in a manner similar to wild-type parasites.

## Testing the hypothesis

### Re-interpretation of previously published observations of Dko P25/P25 *P. berghei *parasites

If the unified model of ookinete invasion is correct, the Dko P25/P28 *P. berghei *ookinetes observed deep within the midgut epithelium between morphologically normal midgut epithelial cells are actually extracellular parasites, outside the midgut epithelium and within the midgut lumen, attempting to enter the lateral apical membrane of midgut epithelial cells. The significant invagination of the midgut epithelium that occurs during parasite entry into midgut epithelial cells creates the appearance that these ookinetes are in intercellular locations within the midgut epithelium. This would be similar to the phenotype recently reported for *P. berghei *ookinetes in which the *maop *gene has been knocked out [57]. Ookinetes lacking MAOP are unable to rupture the apical plasma membrane of midgut epithelial cells [57]. Consequently, although MAOP-deficient ookinetes invaginate the midgut epithelium, these parasites are unable to enter into midgut epithelial cells and remain extracellular embedded against the apical surface of the midgut epithelium [57].

The actual extracellular location of Dko P25/P28 ookinetes apparently "within" the midgut epithelium is also suggested by the presence of unmelanized parasites in a refractory line of *Anopheles gambiae *mosquitoes [27]. Unmelanized parasites were observed apparently deep within the midgut epithelium exhibiting an abnormal gelatinous appearance suggested to result from exposure to either epithelial cell defence reactions or an early stage of the melanisation reaction [27]. However, as mentioned above, most Dko P25/P28 parasites do not appear to induce the epithelial cell defence reactions triggered by invading wild-type parasites [45]. Furthermore, the refractory *An. gambiae *line melanises wild-type parasites after their passage through midgut epithelial cells into the basolateral extracellular space between adjacent midgut epithelial cells [55,58,59]. Consequently, an alternative interpretation is that Dko P25/P28 ookinetes are unmelanized because of their extracellular location against the apical surface of the midgut epithelium, which fails to expose them to either epithelial cell or melanisation immune responses triggered by wild-type parasites. The gelatinous appearance of unmelanized parasites could be explained by prolonged exposure of ookinetes delayed in the process of midgut epithelium entry to the environment of the midgut lumen; for example, prolonged exposure to the mosquito digestive proteases secreted into the midgut lumen. Dko P25/P28 parasites have been shown to be significantly more susceptible to protease digestion *in vitro *than wild-type parasites [27].

However, there is also evidence that some Dko P25/P28 ookinetes do enter the midgut epithelium. A minority of Dko P25/P28 ookinetes are found within midgut epithelial cells, which exhibit the re-distribution and up-regulation of SRPN10 associated with invasion by wild-type parasites [45]. Some Dko P25/P28 parasites are also melanized in the refractory *An. gambiae *line [27] implying entry into and passage through midgut epithelial cells to the basal surface of the midgut epithelium. Further, Dko P25/P28 parasites induce transcriptional up-regulation of mosquito immune response genes, defensin and GNBP, associated with midgut invasion by wild-type parasites [27]. These immune response genes are not induced by transgenic *ctrp*-disrupted *P. berghei *parasites that are unable to invade midgut epithelial cells [27,60]. Again, this implies that at least some Dko P25/P28 parasites successfully invade the midgut epithelium and trigger mosquito immune responses.

### Experimentally testable predictions of our interpretation

There are several experimentally testable predictions that follow from the alternative interpretation for the previous observations of Dko P25/P28 *P. berghei *ookinete invasion of the midgut epithelium outlined above.

First, all melanized Dko P25/P28 parasites in the refractory *An. gambiae *line should be associated with morphologically abnormal midgut epithelial cells – cells through which these parasites have migrated intracellularly – exhibiting protrusion into the midgut lumen, and up-regulation of NOS and SRPN10. In contrast, unmelanized parasites should not be associated with any morphologically abnormal midgut epithelial cells, as these parasites have failed to enter the midgut epithelium and invade midgut epithelial cells. Unmelanized parasites are, however, expected to reside deep "within" the midgut epithelium in apparently intercellular locations between morphologically normal midgut epithelial cells (assuming that ookinetes on the apical surface of the midgut epithelium cannot be melanized). If Dko P25/P28 ookinetes do migrate across the midgut epithelium *via *a solely intercellular route there is no known reason why these parasites should not also be melanized in the basal region of the midgut epithelium. Consequently, if solely intercellular migration does occur melanized parasites should be found that are not associated with any morphologically abnormal midgut epithelial cells.

Second, there should be a quantitative relationship between the density of Dko P25/P28 ookinetes associated with morphologically abnormal midgut epithelial cells and the number of oocysts that subsequently develop on the basal surface of the midgut epithelium. Specifically, the number of oocyst stage parasites should be equal to or less than the number of Dko P25/P28 ookinetes associated with morphologically abnormal midgut epithelial cells, as only ookinetes migrating intracellularly are predicted to become oocysts. The Dko P25/P28 ookinetes located between morphologically normal midgut epithelial cells are not expected to transform into oocysts, as these parasites do not enter, and hence cross, the midgut epithelium. The number of ookinetes apparently migrating *via *a solely intercellular route greatly exceeds the number of intracellular ookinetes [45]. Consequently, if Dko P25/P28 ookinetes do migrate across the midgut epithelium *via *a solely intercellular route, the number of oocysts should greatly exceed the number of ookinetes migrating *via *an intracellular route (i.e. those associated with morphologically abnormal midgut epithelial cells).

## Implications of the hypothesis

The re-interpretation presented here of previously published work on Dko P25/P28 *P. berghei *parasites implies that there are two sub-populations of Dko P25/P28 ookinetes, neither of which migrate across the midgut epithelium *via *a solely intercellular route (Figure 2). A major sub-population of Dko P25/28 ookinetes is unable to penetrate into midgut epithelial cells and remains extracellular within the midgut lumen, outside but embedded against the invaginated apical surface of the midgut epithelium. Consequently, these parasites appear to be in intercellular locations deep within the midgut epithelium, between the lateral membranes of adjacent midgut epithelial cells. It is proposed that these parasites fail to elicit mosquito immune responses triggered by intracellularly invading parasites, are not melanized in refractory *An. gambiae *and do not give rise to oocyst parasite stages. These parasites remain surrounded by morphologically normal midgut epithelial cells, which do not exhibit the morphological abnormalities associated with parasites invading intracellularly [45]. A second minor sub-population of Dko P25/28 ookinetes is able to penetrate into midgut epithelial cells, in a manner similar to wild-type parasites. These parasites are proposed to elicit mosquito immune responses, including up-regulation of defensin [27], GNBP [27], NOS and SRPN10 [45], undergo melanization in refractory *An. gambiae *[27], and form the few oocysts observed in Dko P25/P28 infections [27]. Accordingly, the latter parasite sub-population should be associated with midgut epithelial cells exhibiting morphological abnormalities associated with intracellular invasion by wild-type parasites [45]. However, if loss of P25 and/or P28 prevents entry into midgut epithelial cells, intracellular movement between multiple adjacent epithelial cells may also be inhibited in Dko P25/P28 parasites.

The reason for the existence of the two distinct sub-populations of Dko P25/P28 *P. berghei *ookinetes is unknown. One explanation is that loss of P25 and/or P28 impedes, but does not entirely prevent, penetration of the apical plasma membrane of midgut epithelial cells. Consequently, the entry of Dko P25/P28 ookinetes into the midgut epithelium may be protracted, prolonging the period of exposure to the hostile environment of the midgut lumen, which results in the death of most parasites before completion of midgut epithelial cell penetration. This interpretation is consistent with the observation of lysed Dko P25/P28 parasites on the luminal side of the midgut epithelium [45].

In summary, the unified model of the route of ookinete invasion across the mosquito midgut epithelium suggests a novel, previously unrecognized, function for P25 and/or P28 in mediating ookinete entry into the midgut epithelium. Specifically, the interpretation presented implies that the loss of P25 and/or P28 greatly impairs, but does not entirely abolish, ookinete entry into midgut epithelial cells and probably has relatively little effect on the ability of ookinetes to traverse through the cytoplasm of midgut epithelial cells. A role for P28 in parasite entry into the midgut epithelium is suggested by the deposition of this molecule at the site of ookinete penetration into midgut epithelial cells [44,45]. This interpretation contrasts with the original studies of Dko P25/P28 parasites, which concluded that P25 and P28 do not play a critical role in recognition, attachment or penetration of the luminal surface of the mosquito midgut epithelium [26,27] and suggests that one mode of action of transmission-blocking antibodies to these ookinete surface proteins is to inhibit parasite entry into midgut epithelial cells, as previously hypothesized [8].

## List of Abbreviations

CTRP = circumsporozoite and thrombospondin-related anonymous protein-related protein; Dko P25/P28 = double knockout of P25 and P28; GNBP = gram-negative binding protein; MAOP = membrane-attack ookinete protein; NOS = nitric oxide synthase; Sko P25/P28 = single knockout of either P25 or P28; SRPN10 = serine protease inhibitor 10.

## Authors' contributions

LAB wrote the manuscript and prepared the figures. LRC revised the manuscript. Both authors read and approved the final version of the manuscript.
